# Mn-Based Methacrylated Gellan Gum Hydrogels for MRI-Guided Cell Delivery and Imaging

**DOI:** 10.3390/bioengineering10040427

**Published:** 2023-03-28

**Authors:** Sílvia Vieira, Paulina Strymecka, Luiza Stanaszek, Joana Silva-Correia, Katarzyna Drela, Michał Fiedorowicz, Izabela Malysz-Cymborska, Miroslaw Janowski, Rui Luís Reis, Barbara Łukomska, Piotr Walczak, Joaquim Miguel Oliveira

**Affiliations:** 1 3B’s Research Group, I3Bs—Research Institute on Biomaterials, Biodegradables and Biomimetics, University of Minho, Headquarters of the European Institute of Excellence on Tissue Engineering and Regenerative Medicine, AvePark—Parque de Ciência e Tecnologia, Zona Industrial da Gandra, Barco, 4805-017 Guimarães, Portugal; 2 ICVS/3B’s–PT Government Associate Laboratory, 4806-909 Braga/Guimarães, Portugal; 3 NeuroRepair Department, Mossakowski Medical Research Centre, Polish Academy of Sciences, 02-106 Warsaw, Poland; 4 Small Animal Magnetic Resonance Imaging Laboratory, Mossakowski Medical Research Centre, Polish Academy of Sciences, 02-106 Warsaw, Poland; 5 Department of Neurology and Neurosurgery, School of Medicine, Collegium Medicum, University of Warmia and Mazury, 10-082 Olsztyn, Poland; 6 Center for Advanced Imaging Research, Department of Diagnostic Radiology and Nuclear Medicine, University of Maryland Marlene and Stewart Greenebaum Comprehensive Cancer Center, University of Maryland, Baltimore, MD 21201, USA

**Keywords:** gellan gum, cell delivery, manganese, magnetic resonance imaging, injectable hydrogels

## Abstract

This work aims to engineer a new stable injectable Mn-based methacrylated gellan gum (Mn/GG-MA) hydrogel for real-time monitored cell delivery into the central nervous system. To enable the hydrogel visualization under Magnetic Resonance Imaging (MRI), GG-MA solutions were supplemented with paramagnetic Mn^2+^ ions before its ionic crosslink with artificial cerebrospinal fluid (aCSF). The resulting formulations were stable, detectable by T1-weighted MRI scans and also injectable. Cell-laden hydrogels were prepared using the Mn/GG-MA formulations, extruded into aCSF for crosslink, and after 7 days of culture, the encapsulated human adipose-derived stem cells remained viable, as assessed by Live/Dead assay. In vivo tests, using double mutant MBP^shi/shi^/rag2 immunocompromised mice, showed that the injection of Mn/GG-MA solutions resulted in a continuous and traceable hydrogel, visible on MRI scans. Summing up, the developed formulations are suitable for both non-invasive cell delivery techniques and image-guided neurointerventions, paving the way for new therapeutic procedures.

## 1. Introduction

Cell-based therapies hold great potential for the treatment of central nervous system (CNS) diseases. Indeed, the rationale for transplanting stem cells has been already established in small [1] and large [2] animal models.

In this regard, the CNS can be accessed using different approaches, resulting in cell deposition in different areas. Intraparenchymal cell delivery is performed via direct needle injections into the parenchyma, resulting in a spatially precise cell deposition. This route is currently considered the method of choice for a large number of studies aiming for direct cell delivery into the CNS. In this regard, the safety of intraparenchymal cell injection was already confirmed in studies using large animals [3], and also in phase I/II clinical trials [4], for direct cell transplantation into the spinal cord and brain. Delivery of therapeutic agents directly near the vicinities of a stroke poses a great advantage of intraparenchymal cell transplantation [5,6]. However, the hostile environment near the stroke region hampers successful cell transplantation with effective therapeutic effect [7]. To overcome such limitation, cell-laden hydrogels capable of being injected directly into the CNS have been successfully used, showing their potential to promote a pro-survival environment, even near the stroke region [8]. In addition, while significant progress has been made in the CNS field, disseminated or multifocal diseases, where a broad cell distribution is required, pose a particular challenge for cell delivery [9]. Examples of such multifocal disorders include multiple sclerosis or amyotrophic lateral sclerosis (ALS). For the therapy to be effective, cell delivery must be carried to extensive areas of the CNS. Although promising, intraparenchymal delivery is linked with a higher risk when multifocal cell depositions are needed, making room for new, less invasive gateways to the spinal cord [10]. Intrathecal injection is an alternative route that allows a more widespread delivery of cells in the CNS, and it is therefore considered as an attractive access for spinal cord targeting [11]. With this minimally invasive method, it is possible to dispense cell suspensions, or drugs, directly into the cerebrospinal fluid (CSF), leading to a wide distribution within the CNS. Although intrathecal injection is a routine clinical procedure with a low rate of complications, and numerous stem cell delivery studies (both pre-clinical and clinical) strengthened the feasibility of this route [12,13,14], there are still some bottlenecks that hamper the development of this technique. The cells injected into the CSF as a suspension in a buffer are subject to gravitational sedimentation, with their accumulation in cauda equina or removal within circulating CSF [11,15]. One way to avoid these undesirable effects is to embed the transplanted cells within a biomaterial matrix.

In this regard, injectable hydrogel formulations are particularly promising, mainly due to their (i) soft and pliable nature; (ii) easy transport of nutrients and metabolites; (iii) tissue-like fluidity; (iv) ease of fabrication; and (v) appropriate bio-adhesiveness and integration with biological interfaces [16]. After being injected into the CNS, the hydrogel physically supports the encapsulated cells, protects the biological material from a hostile environment, and it can also assure a widespread distribution along the spinal cord when injected in the intrathecal space [17].

Another challenge of CNS direct cell delivery is the correct hydrogel placement and allocation. Particularly, for intraparenchymal injections, it is hard to predict the hydrogel placement, as well as a possible dilution in the CSF, because the injection is performed into a fluid compartment. Therefore, the possibility to image and monitor the injection procedure in a real-time and non-invasive way adds significant value to the whole therapeutic approach. Such control can allow the establishment of new procedures that avoid excessive injection, or biomaterial misplacement, assuring its correct positioning. Tomographic techniques, and particularly Magnetic Resonance Imaging (MRI), are the most appropriate for image deep structures, such as the intrathecal space or the parenchyma. Using MRI, it is possible to obtain information about the biomaterial/tissue interface as well as biomaterial placement and biodistribution in both pre-clinical and clinical studies. Another advantage is the non-invasive and radiation-free imaging, thus making MRI one of the most appealing imaging modalities [18].

Visualization of the hydrogel using MRI typically requires hydrogel labelling with a contrast agent. Most frequently used contrasts include iron oxide, gadolinium (Gd), or fluorine in a nanoparticulate form. Although not so popular as other contrast agents, Mn^2+^ ions are increasingly studied and used as a positive contrast [19]. The MRI signal is obtained due to the paramagnetic nature of Mn^2+^, yielding a high contrast on T1-weighted MRI images [20,21]. Manganese presents contrast properties similar to those of the most commonly used Gd. However, its undeniable advantage relies on the fact that it does not accumulate for a long-term period and might be incorporated into the organism or expelled, as it represents one of the necessary microelements [22]. Manganese-based contrasts can not only be used for anatomy analysis but also functional studies [23,24]. Moreover, manganese can be delivered both by systemic and localized routes of delivery [25]. We have previously successfully used Mn^2+^ as a contrast in alginate-based hydrogels. It needs to be stressed though that each hydrogel must be carefully investigated both in terms of its properties and the potential release of manganese ions [26]. Therefore, the concentration of Mn^2+^ must be carefully optimized due to the possible Mn-induced toxicity that might occur when high or repetitive doses of Mn^2+^ are delivered [27,28].

Regardless of the method used to reach the CNS, intraparenchymal or intrathecal, several conditions need to be met to successfully use these methods for delivery of hydrogel-embedded stem cells. The hydrogel needs to withstand the shear forces caused by the injection, but also rapidly cross-link upon reaching the CNS to avoid cell and biomaterial escape to unwanted regions. In this regard, gellan gum (GG) presents great potential as an injectable material [29]. Indeed, this natural polymer has been successfully used for several tissue engineering applications, including intervertebral disc regeneration [30], nanoparticles coating [31], bioprinting of brain-like tissues [32], among others [33]. In its native form, GG is thermo- and ionic-responsive, and therefore fast gelation can occur in situ due to body temperature or the presence of metallic ions in body fluids. Nevertheless, GG needs to be heated up to approximately 50 °C to be water-soluble, making cell encapsulation and delivery a challenging process. Although cells could be mixed with GG near-physiological temperatures, the time required for injection could lead to a decrease in temperature and consequent needle clot, as a result of hydrogel cross-link before injection.

On the other hand, the methacrylation of low acyl GG improves the solubility of this polymer, making it water-soluble at room temperature. Thus, methacrylated GG (GG-MA) can be easily used for cell encapsulation procedures. In addition, GG-MA remains responsive to ionic strength and rapidly cross-links in the presence of metallic ions. Bearing in mind that the CSF is rich in Na^+^, Cl^−^, HCO_3_^−^, K^+^, Mg^2+^, and Ca^2+^ [34], it is highly expected that in situ ionic cross-link occurs upon GG-MA injection. More than triggering the desired in situ cross-linking of the hydrogel, the ionic interaction between GG and metallic ions can be used to incorporate Mn^2+^ within the hydrogel matrix for further MRI tracking. Indeed, it has been shown that Mn^2+^ strongly interacts with GG, specifically at the carboxyl group of the D-glucuronate unit [35,36].

Previous work showed that degradable hydrogel blends formed by GG-MA and hyaluronic acid can be combined with Mn^2+^ to support image-guided intrathecal cell delivery [37]. In turn, this work aims to engineer injectable hydrogels with higher stability that can hold the delivered therapeutic agents for longer periods at the injection site. A slower degradation will also prevent a possible deposition of the material at cauda equina. To that end, GG-MA hydrogels were used in combination with Mn^2+^ ions to prepare traceable and stable hydrogels.

## 2. Materials and Methods

### 2.1. Preparation of Methacrylated Gellan Gum (GG-MA), MnCl_2_, and aCSF Solutions

Methacrylated gellan gum (GG-MA) was obtained as previously described by Silva-Correia et al. [30]. Briefly, a solution of low-acyl gellan gum (Gelzan™ CM Gelrite^®^, Sigma-Aldrich) reacted with glycidyl methacrylate (GMA, 97%, Sigma-Aldrich) overnight at room temperature, with constant control of pH at 8.5 and under vigorous stirring. The reaction products were precipitated by the addition of cold acetone and further purified by dialysis (cellulose membrane, molecular weight cut-off 12 kDa, Sigma-Aldrich) against distilled water, for one week. Then, the obtained GG-MA was frozen at −80 °C, freeze-dried, and the final dry material was stored protected from light, in a dry place, until further use. GG-MA solutions of desired concentrations were prepared by dissolving the dry material in Milli-Q water using gentle agitation. Manganese (II) chloride powder (MnCl_2_ powder, Sigma-Aldrich) was used to prepare the MnCl_2_ aqueous solutions used as a supplement for GG-MA hydrogels. Artificial cerebrospinal fluid (aCSF) was prepared following the composition listed in Table 1, and final pH adjustment to 7.3, with NaOH. For in vitro and in vivo assays, dry GG-MA was sterilized by UV light for 30 min in a laminar flow hood. All other materials and solutions were sterilized by filtration (0.22 µm filter).

### 2.2. Preparation of Mn-Based GG-MA Hydrogels

Hydrogel solutions were prepared by mixing 1% (*w*/*v*) GG-MA with MnCl_2_ solutions to obtain hydrogels with 0.1 or 1 mM of Mn^2+^ and 0.75% (*w*/*v*) GG-MA. A solution of 0.75% (*w*/*v*) of GG-MA was used as control. To better mimic in vivo conditions, further cross-link was obtained by hydrogel interaction with aCSF. For that, Mn-based GG-MA (Mn/GG-MA) hydrogels were poured into cylindrical silicon templates (diameter = 8 mm; height = 2 mm), and cross-linked with an excess of aCSF, added dropwise, for at least 5 min.

### 2.3. Rheological Studies

The rheologic properties of the developed hydrogels were obtained using a Kinexus Pro+ rheometer (Malvern Instruments, UK), with the acquisition software rSpace. Oscillatory tests were performed using stainless steel (316 grade) parallel plates, with an upper measurement geometry plate with 8 mm of diameter, and a 20 mm lower pedestal with roughened finish (to prevent sample slippage and resulting errors on the experiments). Pre-gel solutions of 0.75% (*w*/*v*) GG-MA, and 0.75% (*w*/*v*) GG-MA supplemented with 0.1 mM or 1 mM MnCl_2_ were poured in the lower pedestal. The same formulations were also tested after cross-linking with aCSF and for that purpose, hydrogels with 2 mm height and 8 mm of diameter were prepared beforehand with the silicon templates, as mentioned above. For the oscillatory tests, the Linear Viscoelastic Region was previously determined, and then single-frequency oscillation experiments were performed at 0.1 Hz for 30 min. Shear viscosity was determined by rotational experiments, using an upper measurement geometry cone (40 mm diameter and 4° angle). All experiments were performed at 37 °C, and plots are the average of at least 3 experiments.

### 2.4. Injection Ability Test

The possibility to extrude the Mn/GG-MA pre-gel solutions from a 31 G needle was investigated using in-house injection equipment (Paralab). The measurements were performed with a 10 µL Hamilton syringe (Gastight Syringe Model 1701 RN) coupled with a 31 G needle. The syringe was filled with the Mn/GG-MA solution, as well as aCSF (control). All the materials were extruded using a defined extrusion rate (10 µL/min), and the force used to comply with it was measured and recorded using an appropriate software.

### 2.5. Permeability Studies

Permeability studies were performed using 70 kDa fluorescein isothiocyanate–dextran molecules (Dextran-FITC, Sigma-Aldrich). Dextran-FITC was dissolved in Milli-Q water and added to the Mn/GG-MA hydrogel solutions at a final concentration of 125 µg/mL. As before, hydrogels were placed inside 8 mm discs and further cross-linked with aCSF to mimic the delivery of the hydrogels into the intrathecal space. The resulting gels were incubated at 37 °C in aCSF, with mechanical shaking. At different timepoints, 350 µL of supernatant was retrieved and replaced by the same amount of fresh aCSF. At the last timepoint, hydrogels were mechanically destroyed, centrifuged, and the resulting supernatant was used to calculate the concentration of FITC-labelled molecules that were retained inside the hydrogels, and also to calculate the total amount of dextran-FITC that was initially encapsulated. The fluorescence emission of FITC-labelled dextran was measured in a microplate reader (Gen 5 2.01, Synergy HT, BioTek) using an excitation wavelength of 485/20 nm and an emission wavelength of 528/20 nm. The concentration of dextran-FITC released from the hydrogels was finally calculated using a calibration curve, obtained by the measurement of the fluorescence emission of dextran-FITC solutions of known concentrations.

### 2.6. Degradation Profile

The weight loss profile of the Mn-based GG-MA hydrogels upon incubation in aCSF was used to evaluate their degradation along time. For this, the different hydrogel formulations, previously cross-linked with aCSF, were weighed (initial weight, *m_i_*) and incubated in aCSF at 37 °C. All solutions were supplemented with 0.2% (*w*/*v*) sodium azide (Sigma-Aldrich) to avoid bacterial contamination. After 1, 3, 24, 72, and 168 h, the hydrogels were retrieved, the liquid excess was gently removed, and the final mass of samples was determined (*m_f_*). Equation (1) was applied to calculate the weight loss ratio at each time point.

(1) $$Degradation\;\left(\%\right)=\left(\frac{m_{i}-m_{f}}{m_{i}}\right)\times 100.$$

### 2.7. Manganese Release Profile—Inductively Coupled Plasma-Optical Emission Spectroscopy (ICP)

The Mn^2+^ release profile from the Mn-based GG-MA hydrogels was quantified using inductively coupled plasma-optical emission spectroscopy (ICP; JY2000 2, Jobin Yvon, Horiba). Hydrogel cylinders, with 8 mm diameter and 2 mm height, were incubated in aCSF at 37 °C with mechanical shaking. At defined timepoints (0.5, 1, 5, 24, and 48 h), the aCSF supernatant was collected, dissolved in nitric acid and injected into the ICP equipment. Manganese (λ_em_ = 259.37 nm) concentrations in the aCSF solutions were obtained by comparison with standard solutions, with a detection limit of 5 ppb.

### 2.8. Human Derived Adipose Stem Cells (hASCs) Isolation and Culture

Lipoaspirate samples were collected from abdominal regions of healthy human male and female donors with ages between 18 and 57 years, after informed consent, under established cooperative agreements between the Hospital da Senhora da Oliveira (Guimarães, Portugal) and the 3B’s Research Group—University of Minho. After collection, samples were digested with collagenase NB 6 GMP Grade (Serva), and centrifuged to remove all liquids (50× *g*, 5 min). Then, a new collagenase solution was added to the concentrated adipose tissue at a final concentration of 0.2 U/mL, and incubated for 1 h at 37 °C with shaking. Afterwards, the tissue was washed twice with PBS and centrifuged for 10 min at 250× *g*. Samples were rewashed with PBS, and the cellular pellet was then ready for use. The obtained cell pellet was mixed with MSC growth medium (MSCGM BulletKit, Lonza) and transferred into cell culture dishes. After 48 h, the fibroblast-like cells were selected from the rest of the floating debris, rinsed with PBS, and cultured for the next 7 days or until near confluence. The cells were also immunostained for the MSC typical cell surface markers: CD73, CD90, CD105 (data not shown). ADSC presented positive signal for typical MSC markers as well as negative signal for hematopoietic markers according to ISCT guidelines.

### 2.9. Cell Encapsulation

Human derived adipose stem cells (hASCs) were grown as monolayers as previously described. At passage 3–4, confluent cells were detached from tissue culture flasks using Trypsin (Gibco^®^, Life Technologies, New York, NY, USA), and a cell pellet was formed after centrifugation at 1200 rpm for 5 min. The obtained cell pellet was gently resuspended in Mn-based GG-MA pre-gel solution with 0.75% (*w*/*v*) GG-MA and 0.1 M MnCl_2,_ to a final cellular density of 1 × 10^6^ cells/mL. Solutions of 0.75% (*w*/*v*) GG-MA without MnCl_2_ were also prepared and used as controls. Then, a 10 µL Hamilton syringe (Gastight Syringe Model 1701 RN) coupled with a 31 G needle (length = 35 mm) was filled with the cell-leaden solutions, mounted on a stereotaxic syringe pump, and the pre-gel solutions were injected into aCSF using a 10 μL/min extrusion rate. The resulting cell-laden fibres remained in the aCSF for 5 min, after which the aCSF was replaced by 500 μL of cell culture media. The hydrogels were cultured for 1, and 7 days at 37 °C, in a humidified air atmosphere of 5% CO_2_.

### 2.10. Live/Dead Staining

Live/Dead fluorescence assays were performed after 1 and 7 days of culture. LIVE/DEAD Viability/Cytotoxicity Kit for mammalian cells (ThermoFisher Scientific, Rockford, USA) was used to perform the Live/Dead assay, where Calcein-AM-stained live cells and ethidium homodimer-1 (EthD-1) stain dead cells. At each timepoint, the culture medium was removed, 2 μM of Calcein and 4 μM EthD-1 diluted in PBS were added to each well, and samples were then incubated for 20 min at room temperature (RT), protected from light. After the incubation, cells were visualized in the dark using a fluorescence microscope Cell Observer SD (Carl Zeiss, Jena, Germany) in Z-stack mode. Image acquisition was performed at the Laboratory of Advanced Microscopy Techniques, Mossakowski Medical Research Centre, Polish Academy of Sciences. Cell viability was calculated from the ratio between live and dead cells in each fibre, using the ImageJ software (version 2.0.0-rc-69/1.52p).

### 2.11. Animal Surgeries

All the procedures were performed with the approval of the Ethical Committee (IV Local Committee in Warsaw, 117/2015). Intrathecal injections of Mn/GG-MA hydrogel were performed in double mutant MBP^shi/shi^/rag2 (n = 3) immunocompromised mice. In this regard, animals were anesthetized with 1.5–2% isoflurane in oxygen, and placed in the stereotaxic frame in a concord-like position [38]. A small incision (7–9 mm) was made in the midline at the posterior aspect of the skull, to expose the atlanto-occipital membrane. Then, 10 µL of Mn/GG-MA hydrogel, prepared as described above using 0.1 mM of MnCl_2_, was injected into the intrathecal or intracerebral space with a speed of 10 µL/min using a Hamilton syringe coupled with a 31G needle. After injection, the needle was left in the same place for an additional 1 min and then slowly withdrawn. Afterwards, the skin was sutured, and the animal was placed in the MRI scanner.

### 2.12. Phantom Magnetic Resonance Imaging

Tripilot scan was followed by T1 parametric imaging with the 2D Saturation Recovery Spin Echo Sequence with varying repetition times (TR = 200 ms–8000 ms, TE = 9.5 ms, rare factor = 2, NA = 1, FOV = 75 mm × 75 mm, 5 slices 2.0 mm-thick with 2.0 mm gaps, spatial resolution = 586 µm × 586 µm, scan time~13 min) and T2 parametric imaging with the MSME sequence (TR = 5000 ms, TE = 15 ms–480 ms, NA = 1, FOV = 75 mm × 75 mm, 5 slices 2.0 mm-thick with 2.0 mm gaps, spatial resolution = 586 µm × 586 µm, scan time~8 min).

The relative magnetic resonance signal of T1-weighted images of GG-MA phantoms with different concentrations of manganese ions was analysed using the ImageJ software (version 2.0.0-rc-69/1.52p). Identical regions of interest (ROI) were outlined on MR images and the signal intensity was then measured.

### 2.13. In Vivo Magnetic Resonance Imaging

MR imaging was performed immediately after intrathecal and intracerebral (intraparenchymal) injection of the hydrogel, and 24 h after surgery. For the imaging, animals were anesthetized with isoflurane (1.5–2% in oxygen) and positioned head prone in an MRI-compatible water-heated bed. Body temperature and respiration rate were monitored throughout the study with MRI-compatible probes (SA Instruments, Stony Brook, NY, USA). A 7T MR scanner (BioSpec 70/30 USR, Bruker, Ettlingen, Germany) equipped with a transmit cylindrical radiofrequency coil (8.6 cm inner diameter, Bruker) and a mouse brain dedicated receive-only array surface coil (2 × 2 elements, Bruker) was used, and the structural imaging protocol was performed as previously described [39]. Briefly, a T1-weighted 3D FLASH sequence (TR = 12 ms; TE = 4 ms; flip angle, FA = 18; NA = 10; field of view, FOV = 15 mm × 15 mm × 15 mm, spatial resolution = 117 µm isotropic, scan time~25 min) was implemented. Structural imaging was followed by T1 parametric imaging with the 2D Saturation Recovery Spin Echo Sequence with varying repetition times (TR = 410 ms–8000 ms, TE = 22 ms, rare factor = 4, NA = 3, FOV = 20 mm × 20 mm, 8 slices 0.8 mm thick with no gaps, spatial resolution = 156 µm × 156 µm, scan time ~ 23 min) and T2 parametric imaging with the MSME sequence (TR = 5000 ms, TE = 13 ms–416 ms, NA = 1, FOV = 20 mm × 20 mm, 8 slices 0.8 mm-thick with no gaps, spatial resolution = 156 µm × 156 µm, scan time~8 min).

### 2.14. Statistical Analysis

Results are presented as mean ± standard deviation, when appropriate. When applicable, the experimental data were analysed using a single-factor analysis of variance (one-way ANOVA) to assess the statistical significance of the results, followed by post hoc Tukey tests. Statistical significance was set at a *p*-value of ≤0.05. All statistical analysis was performed using GraphPad Prism version 7.0a.

## 3. Results and Discussion

### 3.1. Mn-Based GG-MA Hydrogels

**Figure 1:**
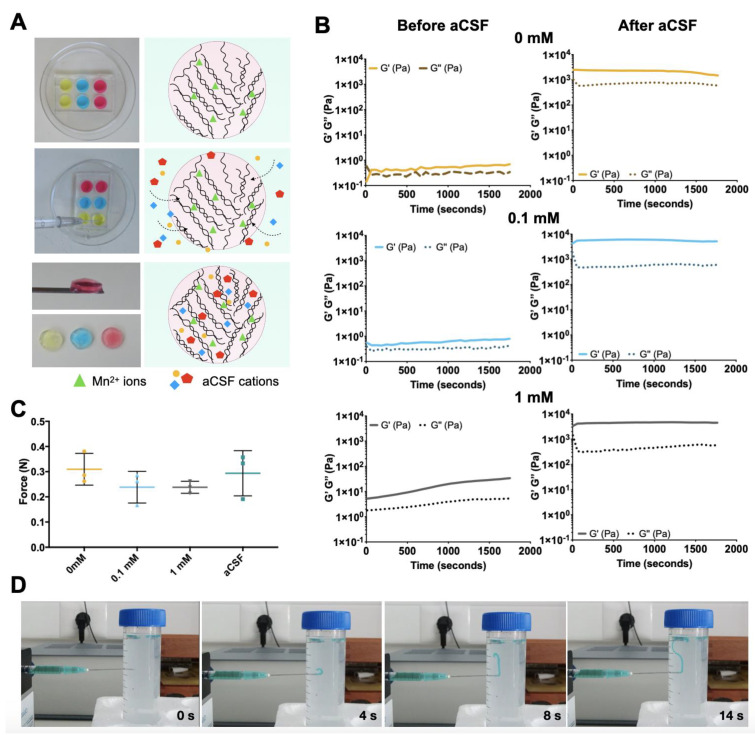
Preparation and rheological characterization of the Mn-based methacrylated gellan gum (GG-MA) hydrogels. (**A**)—Schematic representation of Mn-based GG-MA hydrogel preparation. Pre-gel solutions are poured into silicon templates, followed by the addition of aCSF, resulting in cylindrical hydrogels; (**B**)—oscillatory time sweeps (30 min) of the different hydrogel formulations, before and after addition of aCSF; (**C**) injection force registered after 30 s of hydrogel injection into tube filled with aCSF. Average ± SD, n = 3. (**D**) Time-lapse of hydrogel injection into aCSF. Injection was performed with an 18G needle and with colored hydrogel for better visualization.

Mn-based GG-MA hydrogels (Mn/GG-MA) were prepared using methacrylated gellan gum (GG-MA) as a hydrogel matrix (Figure 1A). Solutions of GG-MA were mixed with MnCl_2_ solutions to obtain the desired final concentration of MnCl_2_ (0, 0.1, and 1 mM), in a final concentration of 0.7% (*w*/*w*) in GG-MA. All formulations were studied by rheological techniques to assess their mechanical properties. Time-sweep curves, plotted in Figure 1B, were performed along 30 min to study a possible gelation effect over time. Particularly, the evolution of the storage modulus (G′), loss modulus (G″), and phase angle (δ) upon the addition of ionic solutions was considered. While G′ is regarded as the stiffness of the material, G″ represents the liquid-like behaviour of the hydrogel. Therefore, these parameters allow the determination of the elastic (G′) and viscous (G″) character of the tested hydrogels, and the ratio between these parcels (δ) providing a quantitative measure of the material mechanical properties [40].

Without the addition of MnCl_2_, 0.75% (*w*/*v*) GG-MA hydrogels showed a very low G′ value of 0.65 ± 0.18 Pa, which was similar to the obtained G″ value (0.35 ± 0.09 Pa). Considering the higher G′ value over G″, one can assume this formulation as viscoelastic solid, as confirmed by the phase angle value of 28.51 ± 4.58 ° that is between the purely elastic (δ = 0°) and purely viscous (δ = 90°) values [41,42] (Supplementary Information, Figure S1). The addition of 0.1 mM MnCl_2_ leads to a modest increase in G′ value to 0.80 ± 0.23 Pa, with G″ following the same trend (0.39 ± 0.10 Pa). In turn, GG-MA hydrogels with 1 mM MnCl_2_ showed a considerably high G′ and G″ values after the 30 min sweep due to the electrostatic interaction of the Mn^2+^ ions with the carboxylic groups of GG-MA. Interestingly, both parameters increased over time, showing a time-dependent interaction between GG-MA polymeric networks and the Mn^2+^ ions. After the time sweep, a G′ and G″ value of 34.54 ± 18.78 Pa and 5.34 ± 1.71 Pa was registered, respectively. As expected, the measured phase angle was lower for hydrogels with 1 mM, 9.69 ± 1.81° (Supplementary Information, Figure S1A), showing that the lag time between strain and shear stress decreases upon addition of MnCl_2_. Hence, one can assume that this formulation has already a “gel-like” character, although with insufficient mechanical stability.

Although the presence of 1 mM MnCl_2_ seems to have changed the rheological properties of the GG-MA hydrogel, with a slight increase in its stiffness, none of the formulations acquired a defined, self-supporting shape. It is known that GG-MA cross-links in the presence of ions, particularly divalent ions such as Mn^2+^. However, the obtained results showed that the ionic concentration used to prepare the hydrogels was not sufficient to overcome the intramolecular electrostatic repulsions of the carboxylic groups in the GG-MA network, thus giving origin to a “weak gel” [43].

The addition of artificial cerebrospinal fluid (aCSF), a salt-rich solution, to the GG-MA led to a more efficient crosslink of the hydrogel network. The ions present in the aCSF can interact with the GG-MA polymer, resulting in a chemical bonding between the divalent ions (e.g., Ca^2+^ or Mg^2+^) and the polymeric network. Additionally, the presence of monovalent ions, such as K^+^ or Na^+^, also contributed to hydrogel cross-link by screening the electrostatic repulsion between the GG-MA ionized carboxylate groups [44]. As a result, the Mn/GG-MA hydrogels are ionically crosslinked by the addition of aCSF. Indeed, after incubation in aCSF for 24 h, the Mn/GG-MA hydrogel acquired a definite shape, possible to be handled. Alongside, G′ values significantly increased to 1463.02 ± 666.19 Pa, 5157.52 ± 2330.58 Pa and 4490.19 ± 1488.04 Pa, for solutions with 0 mM, 0.1 mM and 1 mM, respectively, confirming the increase in the material stiffness upon aCSF-driven crosslinking. An increase in G″ values was also noticed together with the changes in G′. However, significant differences on the phase angle, thus in the ratio G″/G′, were noticed only for GG-MA hydrogels (0 mM MnCl_2_) and hydrogels supplemented with 0.1 mM MnCl_2_, when compared to values without aCSF (Supplementary Information, Figure S1A). Although all formulations showed a viscoelastic nature, the phase angle value was smaller for the GG-MA hydrogels after aCSF incubation, confirming a more elastic behaviour of these hydrogels.

The noticed changes in the rheological properties confirm the feasibility of using Mn/GG-MA hydrogels as in situ cross-linking materials for injectable applications into the CSF. The addition of MnCl_2_ in the concentrations tested was not sufficient to fully crosslink the GG-MA hydrogel, making the formulations suitable for injection. However, when in contact with aCSF, a solution similar to the CSF found in the intraparenchymal space, a self-supporting, defined, viscoelastic hydrogel is formed. Therefore, the CSF can be used to ionically crosslink the Mn/GG-MA hydrogels in situ, without the use of further stimuli.

### 3.2. Injection Ability

From the obtained rheology data, it is possible to infer that the ions present in the aCSF physically cross-link all Mn/GG-MA hydrogel formulations. Yet, it is still necessary to study the hydrogel response to high shear rates, similar to what happens during injection, as well as to measure the force needed to inject the material.

Using steady-state shear measurements, it was possible to observe a decrease in viscosity as shear rate increases, typical of shear-thinning solutions (Supplementary Information, Figure S1B). The non-Newtonian behaviour, noticed for all formulations, strongly suggests the feasibility of hydrogel injection. This capacity to decrease viscosity as a response to increasing shear is highly advantageous for injectable formulations [45].

Regardless, a biomaterial is only clinically relevant for injection if the injection force needed is not damaging to embedded cells and it is feasible in a clinical scenario. Therefore, the force applied to extrude the Mn/GG-MA formulations into aCSF was compared with the force needed to extrude a control solution (aCSF). The assay was performed using 31G needle, as this type of needle size is frequently used for injection in neurological applications, in particular when small animals are used. As plotted in Figure 1C, the pre-gel solutions were easily extruded at low forces: 0.309 ± 0.063 N for GG-MA only; 0.238 ± 0.063 N for 0.1 mM; and 0.238 ± 0.024 N for 1 mM. These values are similar to ones obtained when extruding aCSF, 0.294 ± 0.090 N, thus showing that the tested Mn/GG-MA hydrogels are suitable for non-invasive procedures using injection, even when needles with small diameters are used. No needle clotting was observed during the experiment, and the maximal force used to inject the hydrogel in each experiment was similar for all formulations, with no significant differences observed. Interestingly, after injection into a tube filled with aCSF, the pre-gel solution rapidly cross-links, forming a well-defined fibre structure (Figure 1D). This is particularly important for intrathecal injections, as it shows that Mn-based hydrogels will not disperse within the aCSF due to delayed cross-link leading to undesired stem cell sedimentation.

### 3.3. Hydrogel Permeability

Hydrogel permeability is a key feature for the success of cell-based therapies. Mostly, it is of utmost importance that nutrients and signalling molecules, such as the neurotrophic factors released by transplanted cells, do not face additional diffusional barriers that may hinder their release from the hydrogel to the nervous tissue.

**Figure 2:**
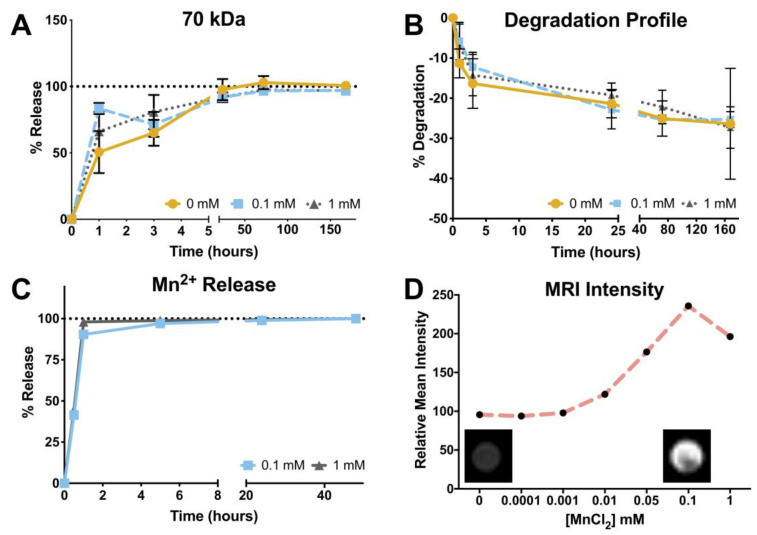
Release and degradation profiles of Mn-based GG-MA hydrogels. (**A**) Release profile of 70 kDa Dextran-FITC, from the hydrogel to surrounding aCSF; (**B**) degradation profile of Mn-based hydrogels along time; (**C**) time-dependent Mn^2+^ release from the prepared hydrogels. Results presented as average ± SD, n = 5. (**D**) relative mean intensity of the magnetic resonance signal of T1-weighted imaging obtained with phantoms of GG-MA hydrogels supplemented with different concentrations of manganese ions (ex vivo).

To study the hydrogel permeability, 70 kDa dextran molecules coupled with FITC were mixed with pre-gel solutions followed by cross-linking and incubation in aCSF for 7 days. As depicted in Figure 2A, for all formulations, most of the dextran molecules were released within the first 2 days of incubation. The release profile of dextran is not affected by the concentration of Mn^2+^ used, as no significant differences were detected between the tested formulations. Thenceforth, it is predictable that molecules within this size range, as most of the key neurotrophic factors released by MSCs, will freely diffuse between the encapsulated cells and the surrounding environment. These include the glial cell-line derived neurotrophic factor (GDNF, 30 kDa), insulin growth factor type-1 (IGF-1, 7.65 kDa), brain-derived neurotrophic factor (BDNF, 14 kDa), and neural growth factor (NGF, 27 kDa), among others, which are of particular importance to the therapeutic effect of MSCs in ALS symptoms [46,47]. Consequently, one can assume that the hydrogels herein proposed will not interfere with the delivery of the aforementioned neurotrophic factors.

### 3.4. Degradation Profile

The degradation profiles of the different hydrogels were also monitored for 7 days while incubated in aCSF, as plotted in Figure 2B. After 1 day of incubation, there was a mass loss of 21.36 ± 3.53% for GG-MA only and 22.86 ± 4.77% and 19.22 ± 3.04% for hydrogels with 0.1 and 1 mM MnCl_2_, respectively. During the following 2 days, mass loss was less evident, and the final hydrogel weight variations were 25.31 ± 1.98% on hydrogels supplemented with 0.1 mM MnCl_2_ and 27.30 ± 5.17% when 1 mM MnCl_2_ was used. Although herein the hydrogels were incubated in aCSF, the obtained degradation profile is aligned with previous studies on GG hydrogel stability in PBS. Different works already pointed out a high stability of GG-based hydrogel, both in the low acyl form or after methacrylation, with a weight loss of only 20% after incubation in PBS for up to 90 days [30,48,49]. When compared with GG-MA only hydrogels, the difference in degradation is not significant, showing again that the introduction of MnCl_2_ ions does not change the GG-MA properties in a substantial way. The obtained formulations are also more stable when compared to the hydrogel blends previously developed, using GG-MA and hyaluronic acid [37]. Therefore, while the blends with hyaluronic acid may be more suitable for strategies requiring the in situ delivery and migration of cells, the Mn/GG-MA hydrogels can be used as cell depots, assuring a correct position of the therapeutic agents for long periods of time. More importantly, the observed stability suggests that Mn/GG-MA hydrogels can hold the delivered cells for long periods, avoiding the undesired sedimentation at cauda equina.

### 3.5. Manganese Release Profile and Magnetic Resonance Imaging

The possibility to perform image-guided interventions is highly appealing, as it can facilitate the precise deposition of the cell-laden hydrogel into the intrathecal space or parenchyma. With that being said, it is expected that the Mn^2+^ ions present in the GG-MA matrix remain in the hydrogel long enough to be detected during the procedure of gel injection. Thus, the Mn^2+^ release profile was obtained via ICP detection. As plotted in Figure 2C, Mn^2+^ ions are quickly released from the hydrogel. Specifically, after 30 min of incubation in aCSF, roughly 50% of the Mn^2+^ ions were released to the surrounding media (45 ± 4% and 46 ± 1% for 0.1 and 1 mM, respectively). The total release was noticed after 1 h of incubation, for both formulations. It was already shown that Mn^2+^ ions interact with the carboxyl group of the D-glucuronate unit of GG [35,36]. However, when in contact with the aCSF, other divalent ions start to diffuse into the hydrogel and to interact with the polymeric network leading to hydrogel crosslink [43,50]. The Mn^2+^ ions are then substituted by other divalent ions, such as Ca^2+^ and Mg^2+^, and consequently released from the hydrogel to the aCSF.

Although the total release of Mn^2+^ was noticed after 1 h of incubation, for both formulations, it should be taken into consideration that Mn^2+^ starts to diffuse only when in contact with the CSF solution. Hence, besides the burst release of the paramagnetic ions, the timeframe until a complete release is achieved is enough for tracking the delivery, placement, and initial biodistribution of the hydrogel within the CSF space. Rapid clearance of the Mn^2+^ contrast signal may be considered advantageous in situations when T1 MRI is further utilized for diagnostic purposes to follow treatment outcomes [51,52].

In order to verify appropriate Mn^2+^ concentration for Mn/GG-MA hydrogel visualization in MRI, phantoms of GG-MA with different concentrations of MnCl_2_ were prepared followed by T1 and T2 weighted MR imaging acquisition (Figure 2D). The MR signal was detected with an MnCl_2_ concentration as low as 0.01 mM. The signal intensity increased with MnCl_2_ concentration, reaching an intensity peak of 0.1 mM, with a 2.4-fold increase in MR signal compared to aCSF. Interestingly, the signal intensity decreased for higher concentrations (1 mM MnCl_2_), which can be attributed to a T2 effect that results in signal loss [20].

The possibility to retrieve an MRI signal with low MnCl_2_ concentrations ensures that, besides the rapid Mn^2+^ release from the hydrogels, the biomaterial is still identifiable by MRI during the transplant procedure. In addition, the use of low Mn^2+^ concentrations prevents possible deleterious effects caused by manganese-induced toxicity [53]. From the obtained results, the formulation using 0.1 mM MnCl_2_ was selected to be used in further studies, as it provides the highest MR signal.

### 3.6. Cell Viability: hASCs Encapsulation

**Figure 3:**
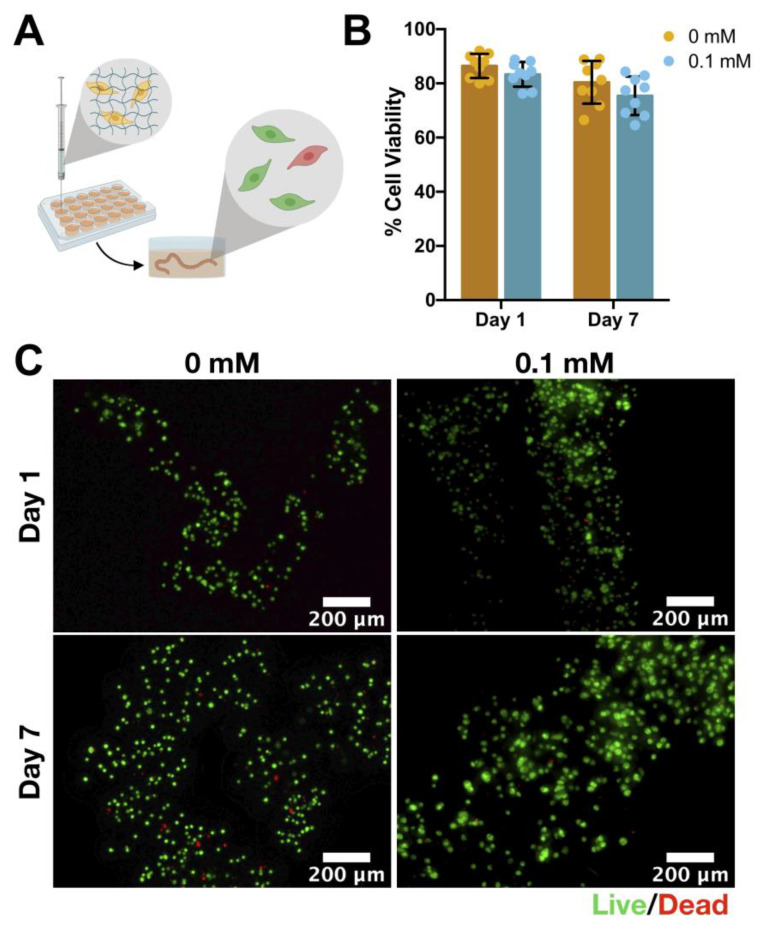
Cell encapsulation within Mn-based GG-MA hydrogel fibers. (**A**) Schematic representation of the experimental outline. Cells were injected directly into aCSF using a Hamilton syringe coupled with a 31G needle, and then cultured for different periods of time; (**B**) cell viability after 1 and 7 days of encapsulation in hydrogels with 0.1 mM MnCl_2_ and GG-MA only (used as control). Results presented as average ± SD, n = 3; (**C**) fluorescence microscopy images of Live/Dead staining after 1 and 7 days of incubation. Live cells showed as green and dead cells as red. Scale bar: 200 μm. Schematic representation created with BioRender.com.

In vitro biocompatibility was studied by encapsulating human-derived adipose stem cells (hASCs) into the developed hydrogels. To better mimic a possible in vivo scenario, hydrogels were extruded into aCSF using a 31G needle, which is typically used for small animal studies (Figure 3A). Considering the aforementioned MRI results, in vitro biocompatibility was assessed using hydrogels prepared with 0.1 mM MnCl_2_, while GG-MA only hydrogels were used as controls. As plotted in Figure 3B, the presence of MnCl_2_ did not significantly impair cell viability along the 7 days of culture. As expected, hydrogels were successfully extruded as fibres, and their shape was maintained throughout the culture time, as depicted in Figure 3C. As a proof-of-concept, 18G needles were also used to extrude hydrogels with 0.1 mM MnCl_2_, envisioning future large-animal studies (Supplementary Information, Figure S2). Similarly, cells remained viable after being encapsulated within the hydrogel matrix, with 92 ± 3% of cells viable 7 days after extrusion. Considering the potential of mesenchymal stem cells [54] and in particular adipose stem cells [55,56] on the treatment of multifocal CNS diseases, the obtained results pave the way to the use of the developed Mn/GG-MA hydrogels as physical support in non-invasive cell-based therapies.

### 3.7. In Vivo Magnetic Resonance Imaging

**Figure 4:**
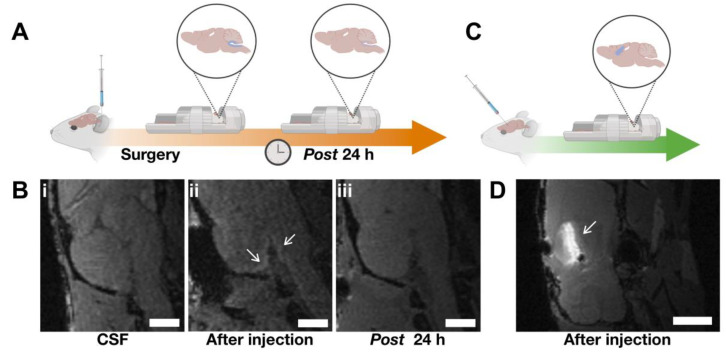
Magnetic resonance T1 weighted imaging of Mn-based GG-MA hydrogels. (**A**) Schematic representation of the in vivo assessment of Magnetic Resonance Imaging (MRI) signal; (**B**) T1 weighted image of (**i**) control MBP^shi/shi^/rag2 mouse; (**ii**) hyperintense signal (arrows) on MR image represent Mn-based GG-MA hydrogel directly after intrathecal transplantation in MBP^shi/shi^/rag2 mice (approximately 20 min after injection); (**iii**) MR scan 24 h after transplantation, where hydrogels are not visible in intrathecal space; (**C**) schematic drawing of intraparenchymal injection and MR scanning; (**D**) MR scan after transplantation of Mn-based GG-MA hydrogel in the parenchyma of MBP^shi/shi^/rag2 mice with prominent hyperintensity (arrow) showing biodistribution of Mn-based GG-MA hydrogel. Scale bar: 2 mm. Schematic representations created with BioRender.com.

The feasibility of using the developed Mn-based GG-MA hydrogels for image-guided cell delivery was further investigated in vivo. For that, hydrogels were injected into the intrathecal space and intraparenchymally in double mutant MBP^shi/shi^/rag2 mice, and followed by MR imaging, as schematically represented in Figure 4A,C.

T1 MRI revealed that hydrogels are visible as a hyperintense signal in comparison to the surrounding tissue (Figure 4(B-ii), arrows) directly after transplantation, confirming the in vitro MR results. However, none of the injected GG-MA hydrogels could be visualized 24 h post-transplantation (Figure 4(B-iii)), which is in accordance with the Mn^2+^ release profile previously discussed. Indeed, the CSF circulation in the intrathecal space is likely responsible for the elution of the Mn^2+^ ions, thus decreasing the hyperintense signal with time. Hence, the engineered hydrogel can be a potential tool for the correct placement of cells along the spinal cord, using a considerably lower amount of MnCl_2_ as compared to other Mn-based hydrogels [20]. As showed before, the MnCl_2_ concentration used to prepare the hydrogels is not deleterious for cells and the rapid release to the CSF prevents a local accumulation of Mn^2+^ ions, which might be harmful to the local neuronal cells, and induce Mn-related toxicity [28]. Mn/GG-MA hydrogels were also visible when hydrogels were injected directly into the parenchyma (Figure 4C,D). MRI-guided intraparenchymal cell delivery is also feasible using the designed hydrogel system, which might be useful for stem cell delivery after a stroke event [8]. As shown in Figure 4D, the MR signal was more intense when the hydrogel was injected into the parenchyma. This shows the importance of the CSF on the clearance of the Mn^2+^ from the hydrogel, since in the parenchyma, the ionic diffusion between the hydrogel and the fluid is reduced, thence increasing the signal. Therefore, one should be aware that Mn^2+^ concentration must be optimized for each of the intended cell delivery routes for better imaging outcomes.

## 4. Conclusions

The present work aimed to design a new stable and traceable hydrogel for image-guided intrathecal cell delivery. By taking advantage of the inherent affinity of methacrylated gellan gum to divalent ions, it was possible to incorporate paramagnetic Mn^2+^ ions within the hydrogel network. Such Mn^2+^ labelled hydrogels can then be used for MRI-guided injection to visualize their biodistribution. The resulting hydrogel was easily injectable, forming distinct, stable, and biocompatible fibres upon injection in a simulated cerebrospinal fluid environment. While the hydrogel formulation would prevent cell removal due to sedimentation or CSF circulation, Mn^2+^ allows real-time visualization of hydrogel position, avoiding an incorrect placement, as evaluated by in vitro and in vivo MR imaging. Thus, the Mn-based methacrylated gellan gum hydrogels developed herein hold great potential on minimally invasive intrathecal cell delivery, tackling two of the main drawbacks reported to date, and possibly open up other applications as a cell/drug delivery matrix for neurological applications when imaging guidance is required.

## Figures and Tables

**Table 1:** Composition of artificial cerebrospinal fluid (aCSF) solution.

	Concentration (mM)
NaCl	125
KCl	2.5
MgCl_2_·6H_2_O	1
NaH_2_PO_4_	1.25
CaCl_2_·2H_2_O	2
NaHCO_3_	25
Glucose	25

## Data Availability

The data presented in this study are available on request from the corresponding author.

## Supplementary Materials

The following supporting information can be downloaded at: https://www.mdpi.com/article/10.3390/bioengineering10040427/s1, Figure S1: Rheologic characterization of Mn-based hydrogels; Figure S2: Cell encapsulation in GG-MA hydrogel supplemented with 0.1 mM.
