# Supplementary material for: Mn-Based Methacrylated Gellan Gum Hydrogels for MRI-Guided Cell Delivery and Imaging
Source: Bioengineering (Basel). 2023 Mar 28;10(4):427. doi: 10.3390/bioengineering10040427 (PMC10135712; doi:10.3390/bioengineering10040427)
Supplement: Supplementary file 1 [file bioengineering-10-00427-s001.zip › bioengineering-2216210-supplementary.pdf]

Supporting Information

Mn-Based Methacrylated Gellan Gum Hydrogels for MRI-Guided Cell Delivery and Imaging

Sílvia Vieira, Paulina Strymecka, Luiza Stanaszek, Joana Silva-Correia, Katarzyna Drela, Michał Fiedorowicz, Izabela Malysz-Cymborska, Miroslaw Janowski, Rui Luís Reis, Barbara Łukomska, Piotr Walczak and Joaquim Miguel Oliveira *

1. Rheological Characterization: Phase Angle and Shear Viscosity

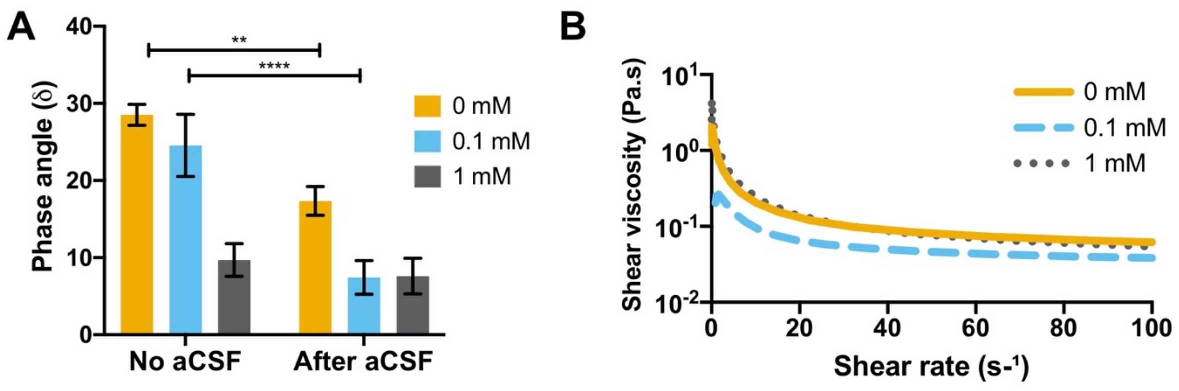

Figure S1 - Rheologic characterization of Mn-based hydrogels. A – Hydrogels phase angle before and after addition of aCSF. Results presented as average±SD of the last registered minute, of at least 3 different samples; ** represents statistical difference between sample of GG-MA only hydrogels (0 mM) before and after addition of aCSF with $p<0.0022$; **** represents statistical difference between sample of GG-MA hydrogels supplemented with 0.1 mM $MnCl_2$, $p<0.0001$; B – Shear viscosity of the different hydrogel formulations, along a shear rate ramp. Shear viscosity drastically decreases upon increasing on the shear rate. Results presented as average, n=4.

2. Biocompatibility: Injection with 18G needle

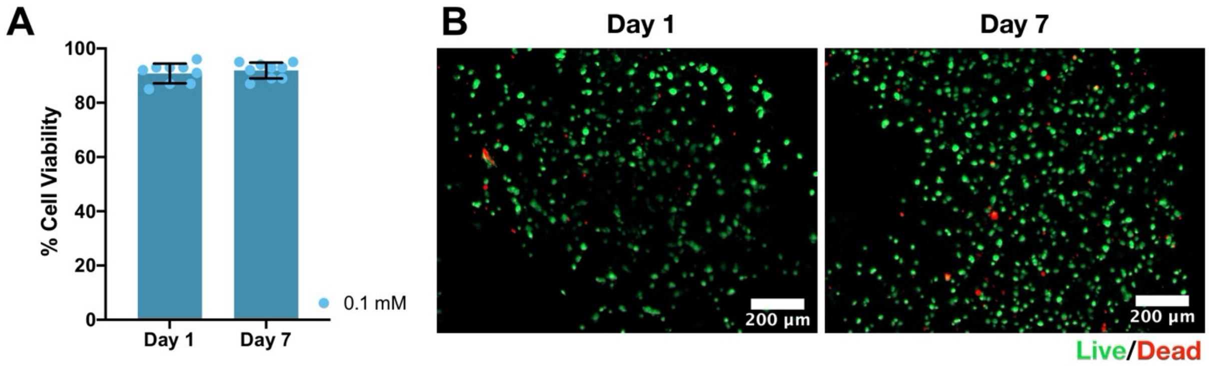

Figure S2 - Cell encapsulation in GG-MA hydrogel supplemented with 0.1 mM. A- Cell viability after 1 and 7 days of culture (average±SD, n=3); B - Fluorescence microscopy images of Live/dead staining after 1 and 7 days of incubation. Live cells showed as green and dead cells as red. Scale bar: 200 µm.
